# Antagonism between Notch and bone morphogenetic protein receptor signaling regulates neurogenesis in the cerebellar rhombic lip

**DOI:** 10.1186/1749-8104-2-5

**Published:** 2007-02-23

**Authors:** Robert P Machold, Deborah Jones Kittell, Gordon J Fishell

**Affiliations:** 1 New York University School of Medicine Smilow Neuroscience Program Department of Otolaryngology 522 First Avenue New York, NY 10016, USA; 2 New York University School of Medicine Smilow Neuroscience Program Department of Cell Biology 522 First Avenue New York, NY 10016, USA

## Abstract

**Background:**

During the embryonic development of the cerebellum, neurons are produced from progenitor cells located along a ventricular zone within dorsal rhombomere 1 that extends caudally to the roof plate of the fourth ventricle. The apposition of the caudal neuroepithelium and roof plate results in a unique inductive region termed the cerebellar rhombic lip, which gives rise to granule cell precursors and other glutamatergic neuronal lineages. Recently, we and others have shown that, at early embryonic stages prior to the emergence of granule cell precursors (E12), waves of neurogenesis in the cerebellar rhombic lip produce specific hindbrain nuclei followed by deep cerebellar neurons. How the induction of rhombic lip-derived neurons from cerebellar progenitors is regulated during this phase of cerebellar development to produce these temporally discrete neuronal populations while maintaining a progenitor pool for subsequent neurogenesis is not known.

**Results:**

Employing both gain- and loss-of-function methods, we find that Notch1 signaling in the cerebellar primordium regulates the responsiveness of progenitor cells to bone morphogenetic proteins (BMPs) secreted from the roof plate that stimulate the production of rhombic lip-derived neurons. In the absence of Notch1, cerebellar progenitors are depleted during the early production of hindbrain neurons, resulting in a severe decrease in the deep cerebellar nuclei that are normally born subsequently. Mechanistically, we demonstrate that Notch1 activity prevents the induction of Math1 by antagonizing the BMP receptor-signaling pathway at the level of Msx2 expression.

**Conclusion:**

Our results provide a mechanism by which a balance between neural induction and maintenance of neural progenitors is achieved in the rhombic lip throughout embryonic development.

## Background

The mammalian cerebellum develops from neural progenitors within dorsal rhombomere 1 (r1) just caudal to the mid-hindbrain boundary and above the opening of the fourth ventricle. In the mouse embryonic brain, closure of the neural tube at around embryonic day 9.5 (E9.5) generally creates a ventricular zone of neural progenitors that give rise to successive waves of differentiating neurons; however, at the opening of the fourth ventricle the neuroepithelium extends directly to the roof plate, resulting in an edge along the cerebellar and hindbrain neural plate (r1–r8) termed the rhombic lip. Located at the caudal boundary of the cerebellar anlage in dorsal r1, the cerebellar rhombic lip is a unique germinal territory that gives rise to granule cells and other neurons of the cerebellum and hindbrain [1-4].

Immediately following neural tube closure, the expression of the mouse Atonal homolog Math1 [5], a basic helix-loop-helix (bHLH) transcription factor that is required for the granule cell lineage and other rhombic lip derived neuronal populations [6-8], begins to be induced in rhombic lip cells that subsequently migrate away from the rhombic lip rostrally over the dorsal surface of the cerebellar anlage [9-11]. The roof plate is required for these events [12-14], and is a source of bone morphogenetic protein (BMP) family members that have been shown to be sufficient to induce cerebellar progenitors to express Math1 *in vitro *[15]. Furthermore, mouse embryos lacking BMP receptor expression in the neural tube lose Math1 expression in the rhombic lip [16], indicating a crucial role for BMP signaling in the ongoing induction of Math1 during rhombic lip neurogenesis.

A variety of fate mapping approaches have led to the conclusion that the cerebellar rhombic lip produces temporally distinct neuronal populations during embryogenesis [11,17,18]. Recently, we have generated a temporal fate map of the Math1 cells of the cerebellar rhombic lip, using transgenesis in mice to label cohorts of Math1 cells by expressing an inducible Cre recombinase (CreER^T2^) under the control of the Math1 enhancer. We and others have reported that, prior to emergence of granule cell precursors, the cerebellar rhombic lip is the germinal origin of specific hindbrain and deep cerebellar neurons [7,8]. Here, we propose that, throughout the peak period of neurogenesis in the rhombic lip (E9.5 to E16.5), there is an ongoing BMP-mediated induction of Math1 in cerebellar progenitors that produces waves of distinct neuronal populations over time. Both the presence of Notch responsive genes in this region [19] and the observation that Notch can antagonize BMP signaling in other neuronal cell types [20] suggest that the Notch pathway may regulate this process. Utilizing both loss- and gain-of-function approaches, we demonstrate that an antagonistic interaction between Notch and BMP receptor signaling in cerebellar progenitors regulates their maintenance and differentiation within the rhombic lip throughout embryonic development.

## Results

### Loss of Notch1 in the cerebellar primordium increases rhombic lip neurogenesis

**Figure 1:**
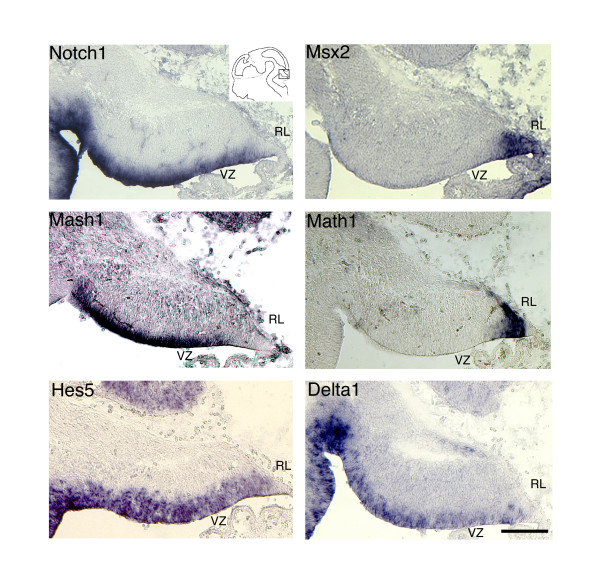
Notch and BMP signaling components in the embryonic cerebellar primordium. Sagittal cryosections of E12.5 wild-type brains were stained by *in situ *hybridization with antisense probes for *Notch1*, *Mash1*, *Hes5*, *Msx2*, *Math1 *and *Delta1*. Differentiating neural precursors in the ventricular zone (VZ) and rhombic lip (RL) are delineated by *Mash1 *and *Math1*, respectively. Msx2 expression is indicative of BMP receptor signaling, and overlaps with the Math1^+ ^territory at this stage. Hes5 and Delta1 are expressed throughout the cerebellar progenitor population, and are generally indicative of high and low Notch1 activity, respectively. The field shown in these panels corresponds to the box in the schematic (inset). Scale bar represents 300 μm.

*Notch1 *mRNA is expressed in the cerebellar primordium as early as E9 (data not shown), and by E12.5 is restricted to neural progenitors in the ventricular zone (VZ) (Figure 1). BMP activity in the rhombic lip and caudal VZ is evident from the expression of Msx2, a homeodomain transcription factor that has been shown to be a target of BMP signaling [21]. Note that the expression pattern of Msx2 overlaps with the expression of Math1 at this embryonic stage (Figure 1). Expression of the bHLH transcription factor Mash1 [22] likely delineates the precursors of Purkinje cells and other GABAergic cerebellar neurons that arise from the ventricular zone [19,23]. Within the VZ, Hes5 expression reflects Notch signaling activity in the cerebellar progenitor pool, while the punctate expression of Delta1 likely indicates a subpopulation of neural precursors that are undergoing differentiation.

**Figure 2:**
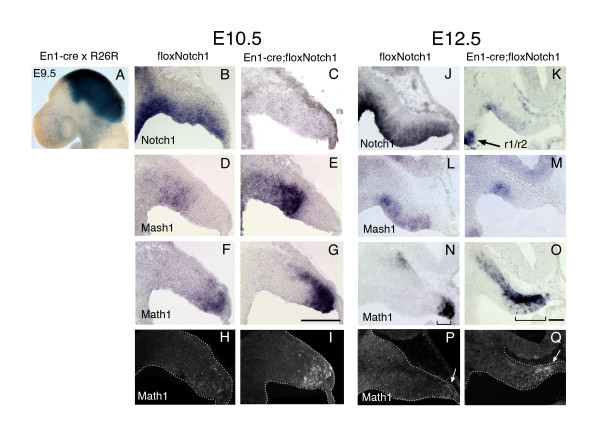
Conditional loss of Notch1 in the early embryonic cerebellum results in precocious differentiation at the expense of the progenitor pool. **(a) **Whole mount β-galactosidase staining of an E9.5 En1cre; Rosa-stopLacZ reveals the extent of recombination across the mid-hindbrain region. **(b-g) ***In situ *hybridization with antisense probes for **(b, c)***Notch1*, **(d, e)***Mash1*, and **(f, g) ***Math1 *on sagittal cryosections of the cerebellar primordia from E10.5 floxNotch1 (b, d, f) and En1cre; floxNotch1 (c, e, g) embryos. **(h, i) **Immunohistochemistry for Math1 on E10.5 floxNotch1 (h) and En1cre; floxNotch1 (i) tissue cryosections. **(j-o) ***In situ *hybridization with antisense probes for **(j, k)***Notch1*, **(l, m) ***Mash1*, and **(n, o) ***Math1 *on sagittal cryosections of the embryonic cerebella from E12.5 floxNotch1 **(j, l, n) **and Engrailed1-cre; floxNotch1 **(k, m, o) **embryos. The r1/r2 boundary is indicated by the arrow in (k). Brackets in (m, n) indicate the extent of *Math1 *induction in the VZ. **(p, q) **Immunohistochemistry for Math1 (white arrows) on E12.5 floxNotch1 (p) and En1cre; floxNotch1 (q) tissue cryosections. Scale bars in (g, o) represent 300 μm.

As a first test for the requirement of Notch signaling in the regulation of rhombic lip neural induction, we examined whether loss of Notch activity in cerebellar progenitors would lead to an increase in rhombic lip neurogenesis as measured by Math1 expression. To circumvent the early (E10) lethality of *Notch1 *null embryos [24], we crossed mice carrying a conditionally null allele of *Notch1 *[25] in which the first exon of the *Notch1 *gene has been flanked by loxP sites ('floxNotch1') with mice expressing cre recombinase under control of the *Engrailed-1 *gene (En1cre) [26]. As shown by β-galactosidase staining in a Rosa26 stop-lacZ background [27], *Engrailed-1 *directs the expression of cre recombinase widely across the mid-hindbrain region by E9.5 (Figure 2a). By E10.5, there is no detectable *Notch1 *mRNA remaining in the cerebellar primordium of the conditional mutant, in contrast to wild-type littermates (Figure 2b, c), with the exception of the most lateral territory, where recombination is incomplete (data not shown). Loss of Notch1 did not have an overt effect on isthmic or roof plate markers at this stage (Additional File 1). Both of the mRNAs for the bHLH transcription factors Mash1 and Math1 are expressed at higher levels in the mutant embryos, but in a complementary pattern: *Mash1 *expression is increased in the rostral cerebellar primordium (Figure 2d, e) whereas there is a robust increase in *Math1 *expression proximal to the rhombic lip in comparison to wild-type littermates (Figure 2f, g). This increase in *Math1 *expression is apparent at the protein level as well (Figure 2h, i), and appears to reflect an increase in both cell numbers as well as the levels of Math1 expression induced within differentiating cells.

By E12.5, mutant embryos exhibit a marked decrease in the size of the cerebellar primordium in comparison to wild-type littermates. Nevertheless, no obvious increase in cell death (terminal deoxynucleotidyl transferase-mediated dUTP nick-end labeling, Additional File 2, or caspase-3 immunohistochemistry), or decrease in proliferation (short term bromodeoxyuridine incorporation) in the residual cerebellar ventricular zone was observed at this stage (data not shown). Following the upregulation of *Mash1 *in the mutants at E10.5 (Figure 2d, e), *Mash1 *expression is decreased in all but the most rostral territory of the ventricular zone in comparison to wild type at E12.5 (Figure 2l, m). In contrast, an increase in *Math1 *expression is still evident in the E12.5 mutants, and scattered *Math1*^+ ^cells are observed in a broader area of the ventricular zone in comparison with sections from wild type littermates at the same medial-lateral position (brackets in Figure 2n, o). This *Math1 *expression pattern in the mutant resembles that observed in the most medial sections from wild-type animals, where the cerebellar primordium is thinner and in closer proximity to the midline and roof plate. The increase in subpial distribution of *Math1*^+ ^cells in the mutant could be accounted for by an increase in migration rate away from the rhombic lip, which is consistent with the observation that Math1 activity is required for subpial migration of rhombic lip neurons [6,28].

**Figure 3:**
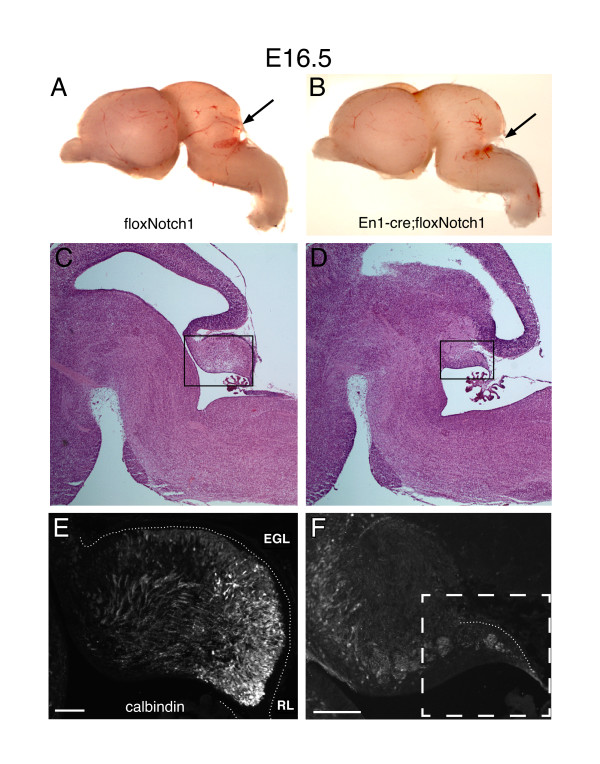
Conditional loss of Notch1 in the cerebellar primordium results in severe hypoplasia and loss of Purkinje cell precursors. **(a, b) **Sagittal whole mount view of E16.5 floxNotch1 (a) and En1-cre; floxNotch1 (b) brains. The arrows in (a, b) indicate the position of the cerebellum. **(c-f) **Sagittal cryosections of floxNotch1 (c, e) and En1-cre; floxNotch1 (d, f) cerebella stained by hematoxylin and eosin histochemistry (c, d) or by immunohistochemistry for calbindin (e, f). Boxes in (c, d) indicate the approximate photographic fields in (e, f). Dashed box in (f) delineates the residual cerebellum in the mutant embryo. Scale bars in (e, f) represent 300 μm.

The increase in *Math1 *expression and concomitant decrease in *Mash1 *from E10.5 to E12.5 in the mutants suggests that early loss of Notch1 results in an increase in rhombic lip neurogenesis at the expense of maintaining the ventricular zone progenitor pool. Consistent with this, by E16.5, the mutant cerebellum is greatly reduced in size in comparison to wild type (Figure 3a–d), and there is a severe decrease in the Purkinje cell precursors (calbindin immunostaining) that are normally generated from the ventricular zone beginning around E12 (Figure 3e, f). In contrast to the results presented in a previous study on the En2-Cre; floxNotch1 mutant mouse [19], we did not observe any postnatal survival of mutant embryos, and even by E18.5 there was no apparent recovery of the mutant cerebellum in terms of size or morphology (data not shown). The greater severity of phenotype in our study most likely results from a more complete loss of Notch1 in the cerebellar primordium using the En1-cre knock-in mouse than was obtained in the En2-cre; floxNotch1 mutant.

**Figure 4:**
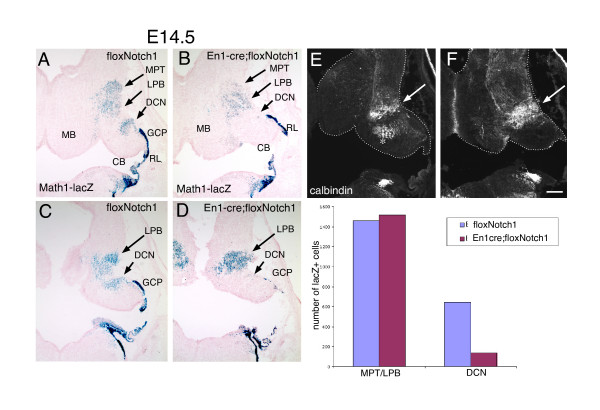
Short term fate map of Math1-lacZ cells generated after conditional ablation of Notch1 in the cerebellar primordium. **(a-d) **Coronal sections from E14.5 floxNotch1 (a, c) and En1-cre; floxNotch1 (b, d) embryos stained for β-galactosidase activity. Long arrows indicate the approximate position of the MPT and LPB neurons, and short arrows indicate the position of the DCN. **(e, f) **Immunohistochemical staining for calbindin on adjacent sections to (a, b) shows an increase in the LPB neuron hindbrain population (white arrows). The asterisk in (e) indicates calbindin positive afferents that are absent in the mutants. Quantification of β-gal^+ ^cells in the control and mutant brains across rostral to caudal tissue sections shows the decrease in DCN in the mutant. Scale bar in (f) represents 300 μm. CB, cerebellum; MB, midbrain; RL, rhombic lip.

Previously, we and others have found that early Math1 rhombic lip neural precursors give rise to specific hindbrain nuclei and the deep cerebellar nuclei (DCN) [7,8], with the peak production of the latter occurring around E11.5, suggesting that these neurons are generated after the majority of hindbrain neurons have been produced (E9.5 to E11.5). To obtain a short term fate map of the *Math1*^+ ^cells that arise in the En1cre; floxNotch mutant cerebellum, we generated this conditional mutant on a Math1-LacZ knock-in (+/-) background [29] and analyzed the residual β-galactosidase staining present in rhombic lip lineages in coronal sections at E14.5. Strikingly, while we observed overall comparable numbers of rhombic lip-derived β-gal^+ ^hindbrain neurons that collectively include the mesopontine tegmental (MPT), parabigeminal (PBG), lateral lemniscus (LL), and lateral parabrachial (LPB) neurons from rostral to caudal positions (Figure 4a–d, long arrows), we observed a dramatic decrease in DCN (short arrows). Neurons of the LPB that arise from a *Math1*^+ ^precursor have been shown to express calbindin embryonically [8] and, interestingly, we observed an increase in calbindin staining in the mutant (Figure 4e, f, white arrows). This result suggests that, in the absence of Notch1 activity, specific early rhombic lip derived lineages are generated in excess at the expense of the hindbrain nuclei and DCN that are specified slightly later.

Surprisingly, we also observed that granule cell precursors (GCPs), a rhombic lip derived population specified after the DCN, appear to be generated to some extent in the mutant, although there is a pronounced decrease in caudal regions (Figure 4c, d). The persistence of granule cells in the mutant may reflect the fact that, while the En1cre driver used in these experiments recombines the vast majority of the mes/r1 primordium, the most lateral regions of the cerebellar primordium escape recombination (data not shown). Thus, it is possible that some granule cells are generated laterally and migrate medially to populate the rostral external granule layer (EGL). Alternatively, as discussed in more depth below, there may be distinct lineage-restricted pools of rhombic lip progenitors that are maintained independently of Notch activity until they begin to divide asymmetrically to produce neurons.

### The level of Notch activity in cerebellar progenitors regulates their cell fate

**Figure 5:**
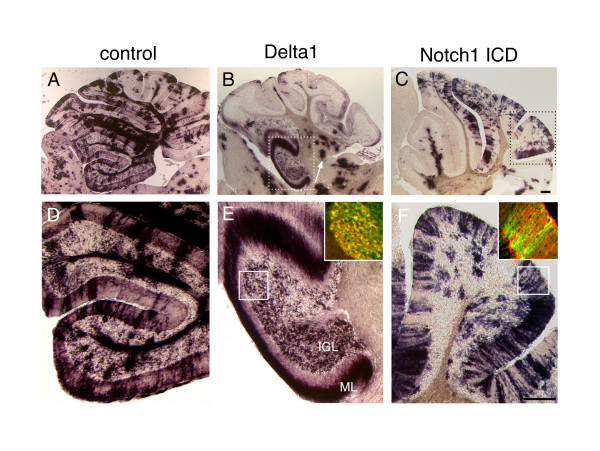
Notch1 activity regulates cell fate decisions in the embryonic cerebellum. Pseudotyped bicistronic retroviruses expressing PLAP and full length Delta1 or the Notch1 ICD were injected into the ventricle of E9.5 embryos *in utero *using ultrasound backscatter microscopy, and the pups sacrificed at P21. Brains of infected animals were cryosectioned sagittally and stained for alkaline phosphatase using NBT/BCIP histochemistry. **(a, d) **Control experiment with retrovirus expressing alkaline phosphatase alone. **(b, e) **Retroviral expression of Delta1 in E9.5 cerebellar progenitors yields granule cells at P21; the inset in (e) shows sections stained by immunohistochemistry for PLAP (red) and Zic2 (green). **(c, f) **Retroviral expression of the Notch1 ICD in E9.5 cerebellar progenitors results in Bergmann glia at P21; inset in (f) shows sections stained for PLAP (green) and BLBP (red). Scale bars in (c, f) represent 300 μm. ICD, intracellular domain; IGL, internal granule layer; ML, molecular layer.

If the loss of Notch1 enhances the responsiveness of cerebellar progenitors to inductive signals that direct rhombic lip neurogenesis, then expression of Notch ligands (for example, Delta) should also render cells more receptive to these developmental cues by virtue of lateral inhibition [30]. To test this hypothesis directly, we generated a pseudotyped bicistronic retrovirus that expresses full length Delta1, along with human placental alkaline phosphatase (PLAP), to allow histochemical detection of transfected cells. Approximately 10^6 ^virions were injected into the ventricle of E9.5 to E10 embryos *in utero *using ultrasound backscatter microscopy [31], and the infected animals analyzed three weeks after birth. Figure 5a shows the results of injections of a control retrovirus expressing only PLAP analyzed at P21 by alkaline phosphatase histochemistry. Infected cells were present in all compartments of the mature cerebellum and did not show an obvious bias towards any particular cell type. Strikingly, similar injections with retroviruses expressing full length Delta1 (Figure 5b) resulted in the labeling of granule cells predominantly, as observed by position, morphology (note the PLAP staining of parallel fibers in the molecular layer), and immunohistochemical co-labeling with antibodies against Zic2 (green) [32] and PLAP (red; Figure 5e inset). Furthermore, Delta1 infections at this stage also appeared to contribute to the DCN (white arrow). Given that these retroviruses require approximately 24 hours to integrate and express the Delta1 protein, we suggest that these Delta infected cells were differentiating and expressing Math1 at around E11.5 to E12 and thus contributed to the DCN and early specified (anterior) GCP that are normally produced at that time.

To examine the effect of constitutive Notch activation in cerebellar progenitors, we performed injections with a retrovirus expressing the intracellular domain of the Notch receptor (Notch ICD), which is known to result in ligand independent activation of the Notch signaling pathway [33]. Injections of Notch1 ICD expressing retroviruses at E9.5 resulted in infected cells developing primarily into Bergmann radial glia (Figure 5c) as shown by their radial morphology and immunohistochemical co-labeling with brain lipid-binding protein (BLBP; red) [34] and PLAP (green; Figure 5f inset). These gain-of-function experiments demonstrate that constitutive Notch activity in cerebellar progenitors prevents these cells from developing as rhombic lip derivatives. While several recent reports have described a role for Notch signaling in *Math1*^+ ^lineages following their specification [35,36], our results argue that, in those contexts, Notch signaling must be regulated in a dynamic manner such that subsequent phases of differentiation can occur.

### Notch1 activity inhibits BMP signaling at the level of Msx1/2 expression

**Figure 6:**
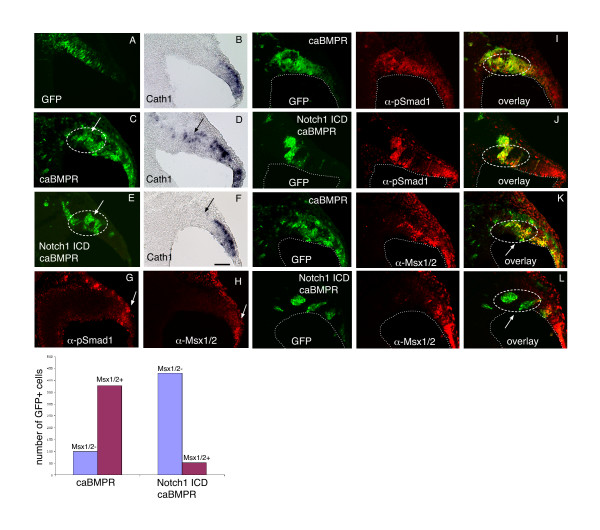
Interaction between Notch and BMP receptor signaling in the cerebellar primordium. **(a-f) **Stage 10 to 12 chick embryos were electroporated *in ovo *with a GFP reporter plasmid and the expression plasmids described below. After two days of incubation, embryos were processed for cryosectioning, and the sections processed by *in situ *hybridization for Cath1. Adjacent sections were photographed for GFP and Cath1 in each experiment. **(a, b) **GFP alone. **(c, d) **GFP and caBMPR1b. **(e, f) **GFP, Notch1 ICD, and caBMPR1b. In the case of caBMPR1b and Notch1 ICD/caBMPR1b electroporations, additional sections were stained by immunohistochemistry for GFP (green) and phosphorylated **(g, i, j) **Smad1 and **(h, k, l) **Msx1/2 expression (red; green and red channels are shown separately to the left of each overlay). The rostral cerebellar primordium is outlined with a dashed oval. In the caBMPR and Notch1 ICD/caBMPR Msx1/2 stainings shown in (k, l), *GFP/Msx1/2+ *and *GFP/Msx1/2- *cells were counted across sections from three electroporated cerebella each and represented as columns. Scale bar in (f) represents 100 μm.

While both Notch and BMP activities have been shown to be crucial during embryonic cerebellar development, how these signaling pathways interact in this context is unknown. To explore the mechanism by which Notch1 signaling in the cerebellar primordium antagonizes the induction of rhombic lip neurogenesis in progenitor cells by BMP signaling, we chose to use the chick *in ovo *electroporation system, which is well suited for short term gain-of-function with multiple expression plasmids [37]. Stage HH 10–12 chick embryos [38] were electroporated at the mid-hindbrain boundary with various expression constructs along with a green fluorescent protein (GFP) reporter plasmid, and analyzed two days later by *in situ *hybridization on cryosections of the cerebellar primordium for changes in the expression of the chick *Math1 *homolog *Cath1 *(Figure 6). To test whether ectopic BMP activity could induce *Cath1 *expression, we electroporated a constitutively active form of the BMP receptor 1b (caBMPR) at a level sufficient to induce patterning changes but not cell death [39,40]. While a control electroporation of a GFP reporter plasmid alone did not induce *Cath1 *expression (Figure 6a, b), ectopic activation of the BMP signaling pathway in the cerebellar primordium (dashed circle) resulted in a robust induction of *Cath1 *expression along the dorsal surface of the cerebellar anlage (Figure 6c, d). Interestingly, when the Notch1 ICD was co-electroporated along with the caBMPR expression plasmid into the cerebellar ventricular zone, the ectopic induction of *Cath1 *was suppressed (Figure 6e, f; Additional File 3). Thus, although ventricular zone progenitors are competent to respond to BMP signaling and express *Cath1*, they are prevented from doing so by high levels of Notch activity.

The observation that Notch activity can block the induction of *Cath1 *by BMP signaling in cerebellar progenitors prompted us to try to determine at what level these pathways intersect within the cell. Electroporations in chick cerebellar primordia of caBMPR alone or caBMPR and Notch1 ICD were stained by immunohistochemistry for phosphorylated Smad1, a direct readout of BMP receptor signaling activity [41]. Smad1 is a transcription factor that, upon phosphorylation by the BMP receptor serine/threonine kinase activity, forms a heterodimer with Smad4 and translocates to the nucleus to activate transcription of target genes (for example, *Msx1/2*; Figure 6h) [42]. As shown in Figure 6, the levels of phosphorylated Smad1 were elevated in both caBMPR (Figure 6i) and caBMPR/Notch1 ICD (Figure 6k) electroporated tissue, demonstrating that expression of the Notch ICD does not interfere at this stage of the BMP signaling pathway. However, while electroporation of caBMPR into the cerebellar primordium (dashed oval) resulted in ectopic expression of Msx1/2 in the ventricular zone (Figure 6j), this induction was suppressed when Notch1 ICD was co-electroporated (Figure 6l). Counts of *GFP*^+^/*Msx1/2*^+ ^cells in the ventricular zone of caBMPR and caBMPR/Notch1 ICD electroporated cerebella from three embryos each are shown in the graph and indicate that, while approximately 80% of ventricular zone cells transduced with caBMPR express Msx1/2, this percentage drops to around 10% when Notch1 ICD is co-transduced. Thus, expression of the Notch1 ICD antagonizes the BMP signaling pathway at the level of Msx1/2 expression.

## Discussion

We have examined the early stages of cerebellar development to gain an understanding of how neurogenesis in the rhombic lip is regulated throughout embryogenesis. We find that Notch signaling is critical for controlling the timing of induction of rhombic lip neurons from the cerebellar progenitor pool as well as for maintaining a progenitor population for subsequent waves of neurogenesis. Using *in vivo *gain-of-function methods, we show that the neural differentiation of cerebellar progenitors can be inhibited by constitutive activation of the Notch1 signaling pathway during early embryogenesis, and that cell autonomous downregulation of Notch activity via expression of Delta1 at early embryonic stages (E11.5) increases the responsiveness of cells to differentiate as rhombic lip neurons. Furthermore, we find that activation of the BMP signaling pathway can induce the rhombic lip proneural gene *Math1 *ectopically in the ventricular zone, and that simultaneous activation of the Notch pathway blocks this inductive effect at the level of Msx expression. Thus, we propose that antagonism between the Notch and BMP signaling pathways regulates the differentiation of cerebellar progenitors throughout the period of neurogenesis in the rhombic lip.

We and others have recently shown that there is an ongoing induction of Math1 in the cerebellar rhombic lip that produces distinct populations of neurons over time; here, we find that this inductive process is regulated by interactions between the Notch and BMP signaling pathways. However, at present little is known about the cerebellar progenitors that give rise to rhombic lip *Math1*^+ ^lineages, and whether they are composed of a number of lineage-restricted progenitor populations or a single pool of progenitors. Previous work from our lab and others suggests that the neural progenitor cells within the ventricular zone are heterogeneous [43-45], and that while at early embryonic stages some progenitor lineages are being maintained by symmetric non-neurogenic divisions, others are becoming neurogenic and divide asymmetrically to produce differentiating neurons. It appears likely that Notch signaling is particularly critical in maintaining a progenitor lineage during asymmetric cell divisions. In this context, our fate mapping results shown in Figure 4 may indicate that there are multiple *Math1*-negative progenitor lineages within the cerebellar progenitor population that give rise to rhombic lip neurons. While the total number of hindbrain neurons (PBG, MPT, LPB) specified appears to be relatively unaffected by loss of Notch1, the LPB neurons appear to be increased in number at the expense of the MPT neurons and DCN, suggesting that these rhombic lip derived neurons may arise from a common progenitor lineage. The persistence of GCPs in this experiment may reflect that there is a distinct progenitor population for GCPs that is maintained independently of Notch activity during the production of hindbrain and DCN until specification of GCPs begins. This possibility could account for the observation that only the early born (rostral) GCPs appear to be specified in the conditional Notch1 mutant, in that Notch signaling would be required to maintain the GCP progenitor pool during the period of GCP induction, and thus these progenitors would be rapidly depleted in the absence of Notch activity. Alternatively, the GCPs that are observed may have arisen from the most lateral regions of the cerebellar primordium that are not recombined by the En1-Cre driver since these cells (but not DCN) are known to migrate from lateral to medial positions [46].

The role of BMP signaling in neural induction has been studied in many contexts, as has the anti-neurogenic role of Notch signaling. However, little is known at present about how these two pathways interact *in vivo *to regulate neurogenesis. A recent study on cell fate determination in neural crest derivatives demonstrated a dominant effect of Notch activation in preventing neuronal differentiation in response to BMP signaling *in vitro *[20]. In this study, it was found that transient Notch activation in neural crest progenitors resulted in a permanent gliogenic fate switch. In the context of the cerebellum, both Notch and BMP signaling have been shown to regulate neurogenesis, but it is not clear that these signaling pathways interact in the same manner as observed in the neural crest. We find it unlikely that cerebellar progenitors that are maintained in the ventricular zone via Notch signaling are committed exclusively to a glial fate. Rather, at this stage of progenitor maturation, Notch signaling acts to inhibit responsiveness to BMP signaling but is not itself instructive until later developmental stages.

Our data demonstrating that the Notch and BMP receptor signaling pathways interact competitively within cerebellar progenitors suggest that the Notch1 ICD and activated Smad1/Smad4 moieties converge on a common target. It has been reported that the Notch1 ICD binds to the core transcriptional activator p300 [47], and forms a complex with p300/CBP-associated protein (P/CAF), Rbp-J and Mastermind like-1 (MAML1) to activate transcription [48]. Recently, it has been shown that phosphorylated Smad1 can be co-immunoprecipitated with the Notch-1 ICD in the presence of p300 and P/CAF [49], suggesting that these core transcription co-activators may mediate the interactions between Notch and BMP signaling. An intriguing complement to the above is suggested by a recent report that Smad1 contains inhibitory phosphorylation sites that are targeted by the mitogen-activated protein kinase (MAPK) signaling cascade [50,51]. Fibroblast growth factor (FGF) signaling from the isthmus could, therefore, potentiate Notch signaling in the cerebellar primordium by decreasing the responsiveness of rostral cerebellar progenitors to BMPs secreted from the roof plate.

The cerebellar rhombic lip is a unique germinal zone that produces specific hindbrain nuclei, DCN, and granule cell precursors in a temporally regulated manner. Our results provide a mechanistic explanation for how the ongoing induction of Math1 in cerebellar progenitors is regulated in the rhombic lip throughout embryogenesis. Because the initiation of neurogenesis in the rhombic lip begins immediately following neural tube closure, and continues late into embryonic development, we find a critical role for Notch1 signaling in the cerebellar primordium during this period to inhibit cerebellar progenitors from responding prematurely to rhombic lip inductive signals. We suggest this represents the first of a set of distinct roles that Notch1 performs in the embryonic cerebellum. We propose Notch1 signaling acts iteratively in the cerebellar progenitor population, first by inhibiting the overproduction of early rhombic lip derived neurons, then by regulating neurogenesis in the ventricular zone [19], and finally by stimulating gliogenesis [20,33]. Furthermore, in addition to Notch1, other Notch family members have been shown to regulate granule cell precursor development during embryogenesis through possible reciprocal interactions with Math1 [36]. Postnatally, Notch2 signaling has been shown to regulate the maturation of granule cell precursors in the EGL by maintaining them in a proliferative state [35]. Thus, it appears that the Notch signaling pathway acts to arrest the differentiation state of cerebellar precursors at multiple developmental stages. Deciphering how the Notch signaling pathway modulates the responsiveness of neural progenitors to developmental cues will be crucial for understanding the regulation of growth and differentiation of the central nervous system throughout embryogenesis.

## Methods

### Mouse genotyping and tissue preparation

Engrailed1-cre, floxed Notch1, Math1-LacZ, and Rosa26 stopLacZ mice were genotyped as previously described [25-27,29]. To generate En1cre; floxNotch1 embryos, the En1-cre line was crossed with homozygous floxNotch1 animals, and the resultant En1-cre; floxNotch1/+ males crossed with homozygous floxNotch1 females. En1cre; floxNotch1; Math1-LacZ animals were generated by crossing En1cre; floxNotch1 (c/+) with Math1-LacZ; floxNotch1 (c/+). The morning of the observed plug was considered day 0.5. Embryos collected at E10.5 to E14.5 were fixed in ice cold 4% paraformaldehyde/phosphate-buffered saline (PBS) for 1 to 2 hours, washed in PBS, and equilibrated in 30% sucrose/PBS overnight. Older embryos and adults were perfused transcardially and the brains dissected prior to sucrose equilibration. For cryosectioning, embryos were mounted in Tissue-Tek OCT (VWR, West Chester, PA) and sectioned at 14 to 20 μM.

### *In situ *hybridization and immunohistochemistry

Section antisense RNA *in situ *hybridization was performed as previously described [52], with the following probes: *Notch1*, *Math1*, *Mash1*, *Msx2*, and *Cath1*. Immunohistochemistry with antibodies against Math1 (rabbit-antiserum; kind gift of J Johnson (UT Southwestern Medical Center), calbindin (rabbit antiserum; Swant, Bellinzona, Switzerland), human placental alkaline phosphatase (sheep α-PLAP antiserum; American Research Products, Belmont, MA, USA), Zic2 (rabbit antiserum; kind gift of J Aruga (RIKEN Brain Science Institute), BLBP (rabbit antiserum, kind gift of T Anthony and N Heintz (Rockefeller University), phosphorylated Smad1 (purified rabbit IgG; Cell Signaling Technologies, Danvers, MA, USA), and Msx1/2 (mouse monoclonal antibody 4G1, ascites, Developmental Studies Hybridoma Bank, Iowa City, IA, USA) was performed as previously described [33].

### Retroviral injections

Preparation, injection, and histochemical analysis of control (CLE) and Notch1 ICD (CLEN) retroviruses have been described previously [33]. Full-length cDNA for human Delta1 was subcloned into pCLE downstream of the EF1α promoter (CLED) and virus prepared as above. We analyzed six to eight P21 brains each for the CLED and CLEN experiments and found three to four for each that had substantial infections in the cerebellum.

### Chick electroporation

*In ovo *electroporation was performed as described previously [40], with the following modifications. Specifically, cDNA for the Notch1 ICD was subcloned into the chick expression vector pMiwIII, such that its expression was directed by the chicken β-actin promoter. The constitutively active BMP receptor 1b and GFP constructs have been described previously [40]. Plasmids were injected into the ventricle at the mid-hindbrain boundary (GFP, 0.2 μg/μl; Notch1 ICD, 1 μg/μl; and caBMPR, 0.33 μg/μl) and two electrodes placed on either side of the neural tube. Five rectangular electric pulses of 15 volts (50 ms each) were then delivered. Embryos were recovered after approximately two days further incubation, fixed for 1 hour in ice cold 4% paraformaldehyde/PBS, washed in PBS, and allowed to equilibrate overnight in 30% sucrose/PBS prior to mounting and cryosectioning. At least three electroporated embryos were analyzed for each experiment.

## Competing interests

The author(s) declare that they have no competing interests.

## Authors' contributions

RPM performed all of the experiments with assistance from DJK while she was a graduate student in the lab of GJF. The manuscript was written by RPM and GJF.

## Supplementary Material

Additional File 1 The E10.5 isthmus and roof plate are not overtly affected by loss of Notch1. Expression analysis of fgf8, wnt1, and otx2 by in situ hybridization on sections of E10.5 control and En1cre;floxNotch1 embryos Click here for file

Additional File 2 Cell death in the cerebellar primordium is not overtly increased at E12.5 upon loss of Notch1. TUNEL staining on sections of E12.5 control and En1cre;floxNotch1 embryos Click here for file

Additional File 3 Notch activation induces hes1 and inhibits cath1 expression in the chick cerebellar primordium. Electroporation of Notch1 ICD in the chick cerebellar anlage results in an increase in hes1 expression and a decrease in cath1 as measured by section in situ hybridization Click here for file
