# Supplementary material for: Antagonism between Notch and bone morphogenetic protein receptor signaling regulates neurogenesis in the cerebellar rhombic lip
Source: Neural Dev. 2007 Feb 23;2:5. doi: 10.1186/1749-8104-2-5 (PMC1820780; doi:10.1186/1749-8104-2-5)
Supplement: Additional File 1 — The E10.5 isthmus and roof plate are not overtly affected by loss of Notch1. Expression analysis of fgf8, wnt1, and otx2 by in situ hybridization on sections of E10.5 control and En1cre;floxNotch1 embryos [file 1749-8104-2-5-S1.pdf]

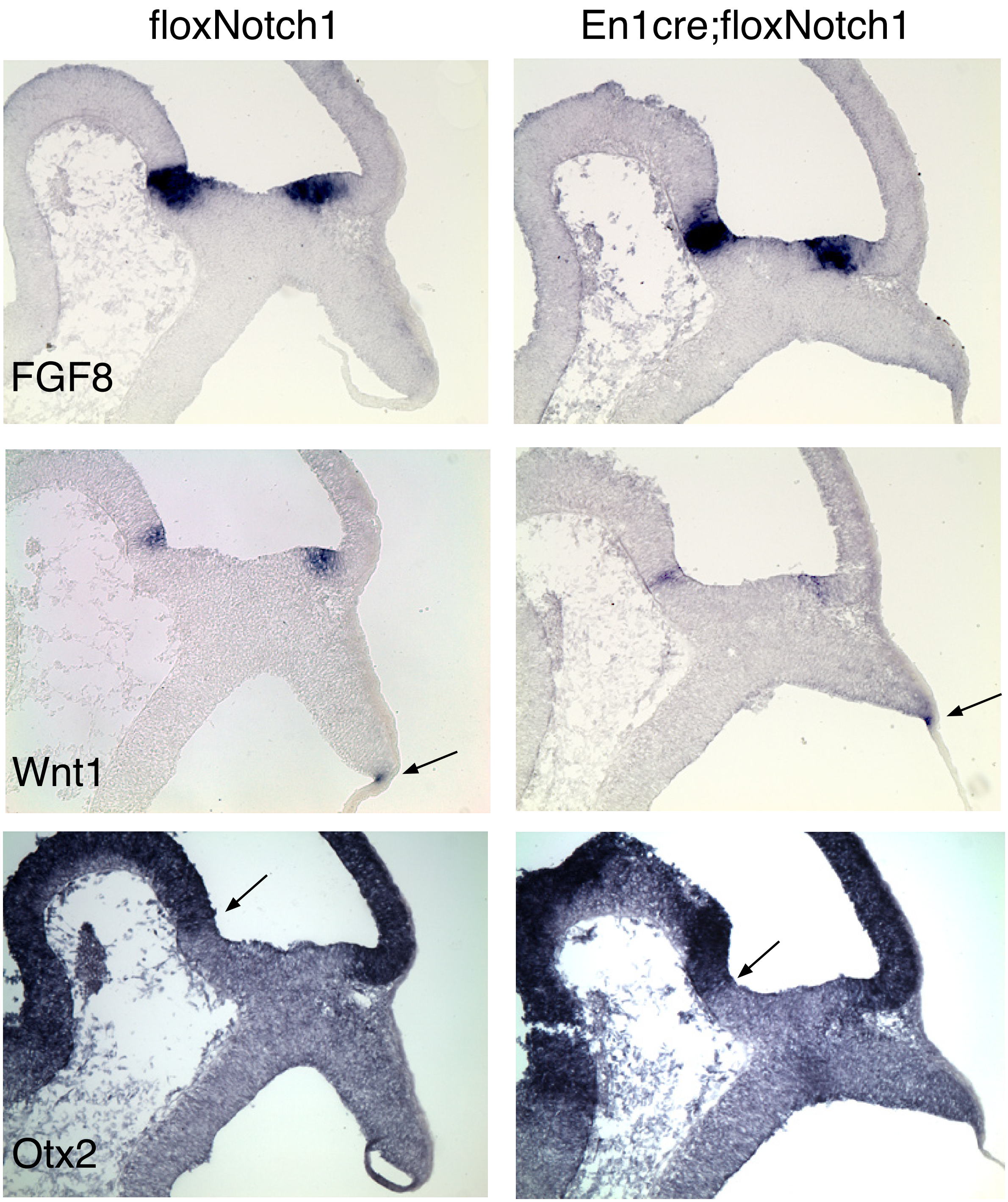

Additional File 1: The roof plate and mid-hindbrain boundary are normal in the En1cre;floxNotch1 conditional mutant. Embryos of floxNotch1 and En1cre;floxNotch1 genotypes were sectioned at E10.5 and analyzed for FGF8 (isthmus), Wnt1 (isthmus/ cb roof plate, arrows), and Otx2 (mesencephalon, arrows) expression by in situ hybridization.
