# Supplementary material for: Antagonism between Notch and bone morphogenetic protein receptor signaling regulates neurogenesis in the cerebellar rhombic lip
Source: Neural Dev. 2007 Feb 23;2:5. doi: 10.1186/1749-8104-2-5 (PMC1820780; doi:10.1186/1749-8104-2-5)
Supplement: Additional File 2 — Cell death in the cerebellar primordium is not overtly increased at E12.5 upon loss of Notch1. TUNEL staining on sections of E12.5 control and En1cre;floxNotch1 embryos [file 1749-8104-2-5-S2.pdf]

TUNEL

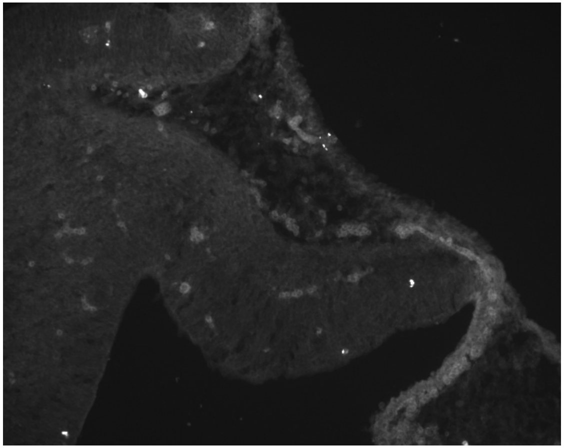

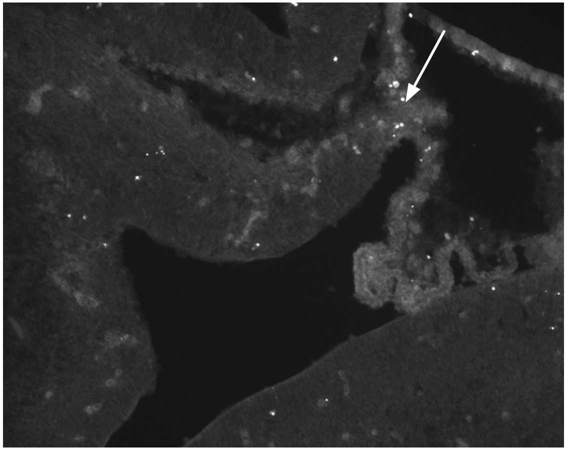

floxNotch1 En1cre; floxNotch1

Additional File 2: Analysis of cell death in the En1cre;floxNotch1 mutant cerebellar anlage. Cryosections were prepared of floxNotch1 and En1cre;floxNotch1 cerebella at E12.5 and assayed for cell death with the TUNEL protocol. No obvious increase in cell death was observed in the mutant cerebellar primordium at this stage, with the possible exception of the medial roof plate (arrow; see also Lutolf et al., 2002).
