# Supplementary material for: Antagonism between Notch and bone morphogenetic protein receptor signaling regulates neurogenesis in the cerebellar rhombic lip
Source: Neural Dev. 2007 Feb 23;2:5. doi: 10.1186/1749-8104-2-5 (PMC1820780; doi:10.1186/1749-8104-2-5)
Supplement: Additional File 3 — Notch activation induces hes1 and inhibits cath1 expression in the chick cerebellar primordium. Electroporation of Notch1 ICD in the chick cerebellar anlage results in an increase in hes1 expression and a decrease in cath1 as measured by section in situ hybridization [file 1749-8104-2-5-S3.pdf]

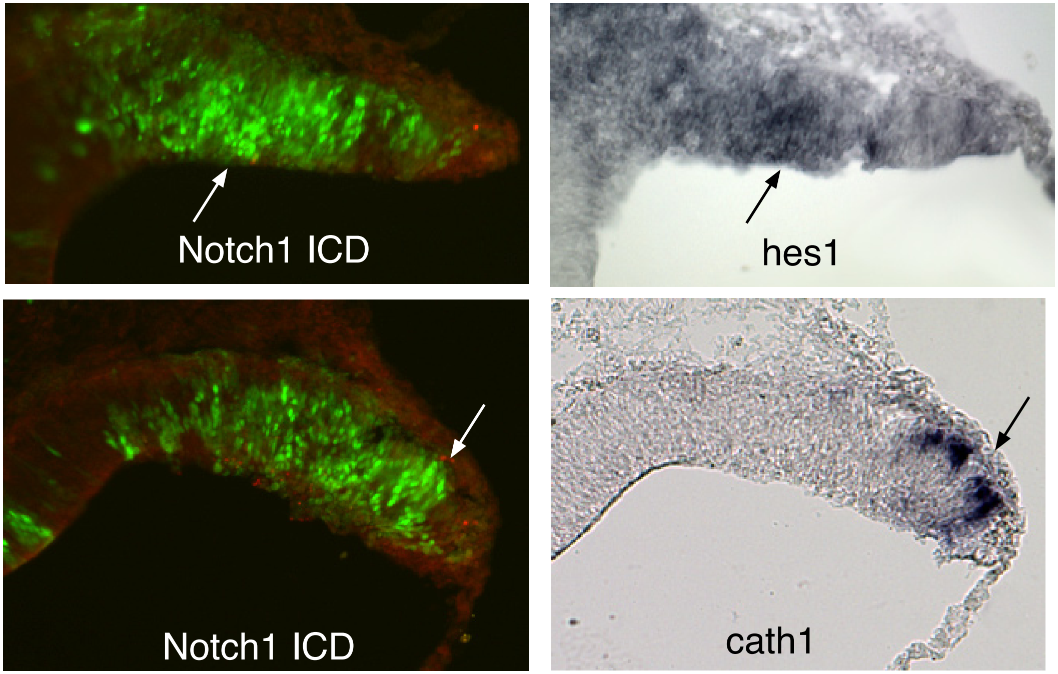

Additional File 3. Activation of the Notch pathway induces hes1 and inhibits expression of cath1 in the chick cerebellar primordium. Stage 10-12 chick embryos were co-electroporated with plasmids expressing GFP and Notch1 ICD, collected after 2 days and cryosectioned for in situ hybridization with hes1 and cath1 antisense probes. GFP expression in the left panel indicates the extent of electroporation; the arrows in both panels indicate a region with ectopic Notch activation where hes1 is induced (top panels) or cath1 expression has been repressed (bottom panels).
